# Equivalent relative biological effectiveness for cell survival and micronuclei formation: insights from a biophysical approach

**DOI:** 10.1002/mp.70040

**Published:** 2025-09-30

**Authors:** Yusuke Matsuya, Ryo Saga, Yidi Wang, Tatsuhiko Sato

**Affiliations:** ^1^ Faculty of Health Sciences Hokkaido University, Kita‐12 Nishi‐5, Kita‐ku Sapporo Hokkaido Japan; ^2^ Nuclear Science and Engineering Center, Research Group for Radiation Transport Analysis Japan Atomic Energy Agency (JAEA), 2–4 Shirakata Tokai Ibaraki Japan; ^3^ Department of Radiation Science Graduate School of Health Sciences Hirosaki University, 66‐1 Hon‐cho Hirosaki Aomori Japan

**Keywords:** biophysical model, microdosimetry, micronuclei, surviving fraction

## Abstract

**Background:**

Micronuclei (MN), which are chromosome fragments, are formed after exposure to ionizing radiation. Radiation‐induced MN is currently used as a quantitative indicator of the chromosomal aberrations detectable at a relatively early phase (e.g., within one cell‐cycle progression). Meanwhile, the MN formation assay is also used to evaluate radiosensitivity (e.g., cell‐killing). As such, the technique to assay the MN formation has been followed with increasing interest. However, the meaning of MN and the corresponding cellular responses remains uncertain.

**Purpose:**

This study presents a biophysical model for estimating MN frequency and theoretically explores the cellular responses associated with MN formation, such as the relationship between MN formation and cell survival.

**Methods:**

We used an integrated microdosimetric‐kinetic (IMK) model that allows the prediction of cell survival after radiation exposure, and we extended the IMK model by introducing a probability of MN formation from lethal lesions by misrepair. To validate the developed model, we estimated the dose, linear energy transfer (LET), and dose‐rate dependencies of MN frequency as well as its relative biological effectiveness (RBE_MN_) and compared them to the corresponding experimental data reported in the literature and measured in this study. The estimation approach of MN frequency from cell survival data and vice versa was also tested.

**Results:**

Our developed IMK model enables the prediction of the MN formation frequency and the RBE_MN_ depending on LET and dose rate for both cancer and normal cells. Comparing the experimental data within this work and the literature, the modeling study clearly shows that radiation‐induced MN is intrinsically related to cell killing after radiation exposure. Our model analyses confirmed that the RBE values for cell survival and MN frequency are equivalent under the same irradiation conditions.

**Conclusions:**

The present model indicates that the analysis of MN is useful in both radiation therapy and radiation protection to quantitatively evaluate curative effects and histological damage at early stages after exposure.

## 1 INTRODUCTION

Ionizing radiation is well known to induce DNA lesions (e.g., DNA double‐strand breaks (DSBs))^1^ as early biological responses and to lead to cell death and chromosomal aberrations^2^ as late responses. In radiation biology and radiation therapy, in vitro and in vivo surviving fractions (in other words, clonogenicity) are measured using a colony assay.^3^ In radiation protection, in 1989, the International Commission on Radiological Protection (ICRP) assumed that cell killing is the sole mechanism of nonstochastic effects.^4^ From such, biological data on the dose–response curve of cell survival plays a key role in determining radiation‐induced biological effects and has been scientifically accumulated to date,^5^ for instance, in the database of the particle irradiation data ensemble (PIDE) for cell survival.^6^ Meanwhile, colony assay takes a long time to form colonies, typically around 1–2 weeks. To address this issue, micronuclei (MN) formation assays are sometimes used for evaluating radiosensitivity.^7^, ^8^ Radiation‐induced MN, which are chromosome fragments, are also currently used as a quantitative indicator of chromosomal aberrations. Whereas the MN are believed to be formed by unrepaired DSBs^9^ associated with cell death, it is also used as a sensitive biological indicator of clastogenic effects.^10^ The technique to assay the MN formation has been followed with increasing interest.^11^ However, the implications of MN formation for radiation protection and therapy remain uncertain.

Radiation‐induced MN can be measured by means of several assays, for example, cytokinesis‐block micro‐nucleus assay (CBMN), mammalian erythrocyte MN assay, etc.^12^ In some experimental papers, the measured MN frequency as a function of absorbed dose is evaluated using the linear‐quadratic (LQ) function.^13^ The LQ model typically and quantitatively evaluates dose–response curves on the surviving fraction of cells in radiation biology and radiotherapy.^3^, ^14^ The model must be fitted to the experimental survival and MN data measured under different irradiation conditions (i.e., linear energy transfer (LET) and dose‐rate dependence) to grasp the radiosensitivities for various exposure situations. For such reasons, the fitting procedures using the LQ model are inefficient and much less suitable for mechanistic studies. To clarify the role of MN and its application to radiosensitivity analysis, it is necessary to develop a theoretical model for predicting the MN frequency based on the scenarios of cellular responses after radiation exposure.

To date, several biophysical models have been developed worldwide,^15^, ^16^, ^17^, ^18^, ^19^ such as the Local Effect Model (LEM),^16^ Biophysical ANalysis of Cell death and chromosome Aberrations (BIANCA),^17^ Nanodosimetry and Oxydative (NanOX),^18^ and the Stochastic MK (SMK) model.^19^ Among the models, to address the above issues, one of the theoretical models considering microdosimetry and DNA damage repair kinetics during and after irradiation, named the integrated microdosimetric‐kinetic (IMK) model,^20^, ^21^ is useful. The IMK model was developed by modification based on the MK model proposed by Hawkins.^22^, ^23^ The ability to estimate the LET and dose‐rate dependencies on cell survival was proved by comparing various experimental data on DNA damage and cell survival.^20^, ^21^, ^24^, ^25^ Particularly, the assumption of potentially lethal lesions (PLLs) and the misrepair processes might be useful for modeling the formation of MN. In general, experimental studies explore the cellular‐response mechanisms by accumulating huge amounts of measured data, while modeling studies can suggest potential scenarios for how cellular responses might be induced based on existing experimental data. In other words, theoretical prediction approaches can efficiently advance the elucidation of cellular response mechanisms.

In this study, we propose a biophysical model for estimating dose, LET, and dose‐rate dependencies of MN formation frequency and its relative value, including the relative biological effectiveness (RBE), by extending the IMK model. Assuming that the misrepair of PLLs forms MN, the developed model enables the exploration of the cellular responses associated with MN formation and the estimation of the RBE values. The RBE is used for evaluating curative effects and radiation risks. Through these model verifications in the comparison with the experimental data of LET and dose‐rate dependencies, including our in vitro work, we show that RBE values for both cell survival and MN frequency are equivalent under the same irradiation conditions. The present model would contribute to the precise understanding of radiation‐induced biological effects in both radiation therapy and protection.

## 2 OVERVIEW OF THE IMK MODEL

This section introduces the IMK model for predicting DNA damage kinetics and the surviving fraction. To date, the model has considered various factors, such as cell recovery (sub‐lethal damage (SLD) repair),^26^, ^27^ LET (microdosimetry),^20^, ^28^ and intercellular signals (bystander effects),^29^, ^30^, ^31^ that affect DNA damage and the surviving fraction, etc. In this study, we used the IMK model, considering microdosimetry and SLD repair, and modified it to predict the MN frequency.

### 2.1 Assumption in the IMK model

In the IMK model, the nucleus of cells is divided into multiple small targets (so‐called domains). The energy deposition for each domain represents specific energy *z* (Gy) defined in microdosimetry.^28^ Although the size of the domain depends on the cell‐line type, the shape is generally assumed to be a simple sphere with a diameter of < 1.0 µm.^32^, ^33^ After irradiation, PLLs are assumed to be induced in a domain containing a DNA amount of *g* (kg) and are proportional to *z* (Gy). The PLLs are assumed to be transformed into lethal lesions (LLs) or repaired at constant rates (*a* and *b*_d_ for an LL induction, and *c* for its repair). In addition to three constant rates, we added the fourth assumption of forming an MN during the transformation of an LL as below:

(i) A PLL may transform into an LL at a constant rate of *a* in h^−1^;
(ii) Two PLLs may interact and transform into an LL at a constant rate of *b*_d_ in h^−1^;
(iii) A PLL may be repaired at a constant rate of *c* in h^−1^;
(iv) An MN may form during the transformation of an LL with a probability of *h*.

**FIGURE 1:**
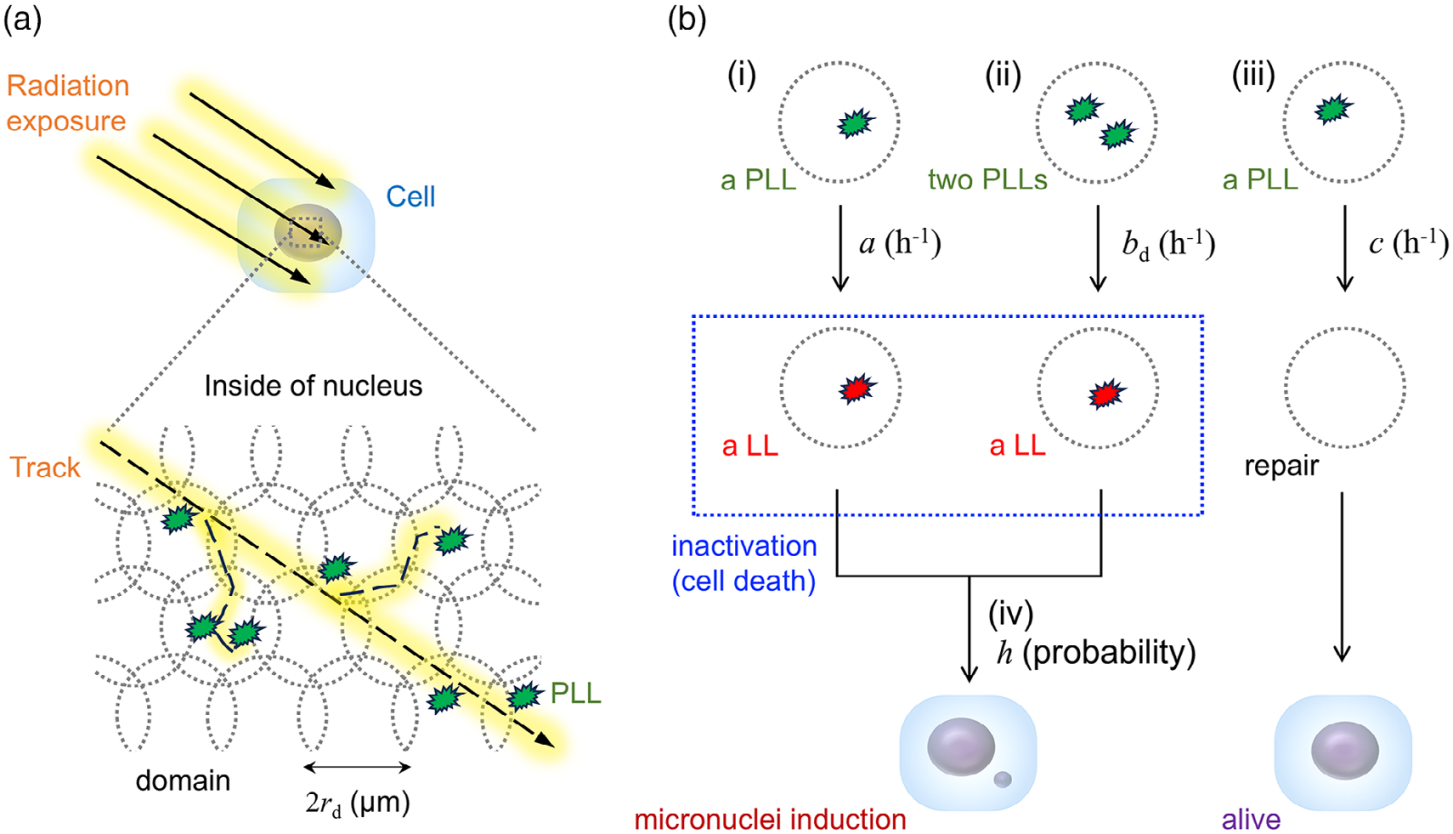
Schematic representation of the assumptions of the IMK model. (a) The cell nucleus is divided into multiple small targets (so‐called domain), and the energy deposition for each domain (specific energy *z* (Gy)) is considered. (b) Transformation of PLLs and LLs is assumed in the model. The fourth assumption of forming an MN during the transformation of an LL is newly introduced in this study. If LLs are completely repaired, the cell can survive radiation exposure. Meanwhile, the MN is formed from LLs with the probability *h*. MN, micronuclei; LLs lethal lesions; PLLs, potentially lethal lesions

The schematic representation of the assumptions in the IMK model in this study is depicted in Figure 1. To date, the domains may be interpreted as interphase chromosome territories,^17^ and PLLs and LLs may be associated with DSBs^17^, ^34^ and unstable chromosome aberrations,^17^ respectively.

### 2.2 Surviving fraction

Based on the assumptions (i)–(iii) described above, the kinetics of PLLs and LLs during and after irradiation are expressed using the rate equations as described previously.^21^, ^24^, ^25^ The rate equations of PLLs and LLs were verified compared to the dynamics of time‐dependent DSBs measured using γ‐H2AX focus formation assay.^24^, ^25^ By solving the rate equations, the relationship between absorbed dose *D* (Gy) and surviving fraction *S* in the consideration of microdosimetric quantity, and SLD repair are described as follows:

$$\begin{array}{cc} -\ln S=<w> & =\left(\alpha_{0}+\frac{y^{*}}{\rho \pi r_{d}^{2}}\beta_{0}\right)\dot{D}T+\frac{2\beta_{0}}{{\left(a+c\right)}^{2}T^{2}}\\ & \;\times \;\left[\left(a+c\right)T+\;e^{-(a+c)T}-1\right]{\left(\dot{D}T\right)}^{2} \end{array}$$

(1) $$=\left(\alpha_{0}+z_{1D}^{*}\beta_{0}\right)D+F\beta_{0}D^{2}=\alpha D+\beta D^{2},$$

where <*w>* is the mean number of LLs per cell nucleus, $\dot{D}$ is the absorbed dose rate (Gy/h), *T* is the dose‐delivery time (h), *α*_0_ and *β*_0_ are the cell‐specific coefficients for the dose (Gy) and dose squared (Gy^2^) to express the number of LLs, respectively, $\alpha =\alpha_{0}+z_{1D}^{*}\beta_{0}$, $\beta =F\beta_{0}$, $z_{1D}^{*}$ = *y* ^*^/ρπ*r*_d_^2^, *y* ^*^ is the lineal energy (keV/µm) considering over kill effect, and ρ and *r*_d_ are the density and the radius of the domains, respectively. The microdosimetric quantity *y* ^*^ can be given by

(2) $$y^{*}=\frac{{y_{0}}^{2}\int \left[1-\exp\left(-y^{2}/{y_{0}}^{2}\right)\right]f\left(y\right)dy}{\int \mathrm{yf}\left(y\right)dy},$$

where *y* is the lineal energy (keV/µm), *f*(*y*) is the probability density of *y*, and *y*_0_ is a so‐called saturation parameter to express the overkill effect.^15^ The y* value can be calculated by using the Monte Carlo track‐structure simulation of Particle and Heavy Ion Transport code System (PHITS).^35^ In addition, *F* corresponds to the Lea–Catcheside time factor,^36^ which is SLD repair traditionally defined in radiation biology.^26^, ^27^

### 2.3 Micronuclei formation

In assumption (iv), an MN forms during the transformation of the LLs with a probability of *h*. By multiplying *h* by the constant rates of *a* and *b*_d_, the number of MN can be expressed by the IMK model. In the conventional MK model, the repair is interpreted as the non‐homologous end‐joining (NHEJ) of DSBs. Thus, the transformation of PLLs to LLs by the constant rates (*a* and *b*_d_) can be assumed as the misrepair. Using Equation (1) and the probability *h*, the MN frequency *F*_MN_ can be expressed as,

(3) $$\begin{array}{c} F_{\mathrm{MN}}=h\;<w>=h\left(\alpha_{0}+z_{1D}^{*}\beta_{0}\right)D+\mathrm{Fh}\beta_{0}D^{2}\\ =\left(\alpha_{m0}+z_{1D}^{*}\beta_{m0}\right)D+F\beta_{m0}D^{2}\\ =\alpha_{m}D+\beta_{m}D^{2} \end{array},$$

where *α*_m0_ and *β*_m0_ are the cell‐specific coefficients for *D* and *D* ^2^ for expressing the frequency of MN, respectively, that is, *α*_m0_ = *hα*_0_ = *hak*_d_<*G*>/(*a**+**c*) and *β*_m0_ = *hβ*_0_ = *hb*_d_ *k*_d_^2^<*G*>^2^/[2*p*(*a**+**c*)], *α*_m _=$\;\alpha_{m0}+z_{1D}^{*}\beta_{m0}$, $\beta_{m}=F\beta_{m0}$. The *k*_d_ is the PLL yield per DNA amount per Gy, <*G*> is the mean amount of DNA per cell nucleus, *p* is the mean number of domains packed in a cell nucleus, (*a*+*c*) is the sum of rate constants of *a* and *c*, which corresponds to the parameters described in Figure 1. To estimate the MN frequency, it is necessary to obtain the *h* parameter and the cell‐specific coefficients (*α*_0_ and *β*_0_). The coefficients for predicting cell survival (i.e., *α*_0_ and *β*_0_) can be obtained by fitting the model (Equation 1) to the experimental survival data after acute irradiation at a high dose rate.

### 2.4 Relative biological effectiveness

The IMK model allows the calculation of the relative biological effectiveness (RBE). When calculating the RBE for cell survival (RBE_SF_), the absorbed dose leading to 10% survival (*D*_10_) is often used,^37^, ^38^ and the acute‐irradiation condition is generally assumed ($\beta \;≅\;\beta_{0}$). Considering these, we used the *D*_10_ and RBE_SF_ expressed as follows:

(4) $$D_{10}=\frac{1}{2\beta }\left\{\sqrt{\alpha^{2}+4\beta S^{*}}-\alpha \right\},$$

(5) $$\mathrm{RB}E_{\mathrm{SF}}=\frac{D_{10(\mathrm{photon})}}{{D_{10}}^{*}},$$

where *S* ^*^ is the negative of the natural log of the surviving fraction *S*, that is, *S* ^*^ = −ln(0.1), *D*_10_(photon) and *D*_10_^*^ are the *D*_10_ value for photon beams and that for any radiation type, respectively.

Meanwhile, based on the previous report,^8^ the RBE for MN frequency (RBE_MN_) is defined as the ratio of coefficient to dose (i.e., $\alpha_{m}$) of certain radiation and that of photon beams as follows:

(6) $$\mathrm{RB}E_{\mathrm{MN}}=\frac{{\alpha_{m}}^{*}}{{\alpha_{m}}_{\left(\mathrm{photon}\right)}}=\frac{h\alpha^{*}}{h\alpha_{\left(\mathrm{photon}\right)}}=\frac{\alpha^{*}}{\alpha_{\left(\mathrm{photon}\right)}},$$

where *α*_m(photon)_ and *α*_m_^*^ are the $\alpha_{m}$ values for photon beams and any radiation type, respectively, *α*_(photon)_ and *α* ^*^ are the *α* values for photon beams and any radiation type, respectively. It is recognized that the RBE value increases with decreasing dose for cells.^39^ Therefore, Equation (6) represents the maximum RBE value for the MN frequency.

## 3 MATERIALS AND METHODS

### 3.1 Estimates of MN frequency from survival data

To check the performance of the developed model, we first determined the model parameters by fitting the model to the dose–response curve of the surviving fraction. We then used the determined model parameters of human salivary gland (HSG) cells and squamous cell carcinoma (SCC) cells for estimating the LET dependence on MN formation. Meanwhile, we also used the determined model parameters of human prostate cancer cell line DU145 and human lung fibroblast WI‐38 cell line for estimating the dose‐rate dependence.

#### 3.1.1 Determination of the model parameters

The model parameters of the HSG and SCC cell lines were determined through the following procedures: (i) the lineal energy distributions and *y* ^*^ values for photons and carbon ions were calculated using the [t‐sed] tally in the PHITS code,^35^ which is the convenient function allowing the rapid computation of the probability density of deposition energies in microscopic sites under any radiation field, using an analytical microdosimetric function, necessary for estimating the surviving fraction after exposure to charged particles^40^; (ii) the parameter sets [*α*_0_, *β*_0_, (*a*+*c*)] were determined by fitting Equation (1) to the experimental survival data^41^, ^42^ obtained from irradiation with photon beams using Markov chain Monte Carlo (MCMC) simulation^43^; (iii) the cell‐specific size of domains (*r*_d_) was determined by comparing the estimated survival with the experimental data for carbon ions (i.e., 290 MeV/n carbon ions with 13, 46, 80, 100 keV/µm).^41^, ^42^ Note that we changed the *r*_d_ value and repeatedly performed the steps (i) to (iii) until obtaining a good agreement with the experimental data on LET dependence. When performing the MCMC simulation in step (ii), we used the previous information on the (*a*+*c*) value of tumor cells.^44^ Note that the energy distributions of 6‐cm SOBP ^12^C ions were reproduced by comparing the dose calculation by PHITS to the measured one^41^ (see Figures S1 and S2).

Meanwhile, the model parameters of the DU145 and WI‐38 cell lines were determined with only biological data after X‐ray irradiation following the approaches described below. As for the WI‐38 cell line, in our previous report, we obtained the model parameters [*α*_0_, *β*_0_, (*a*+*c*)] using the DSB repair curve and dose–response curve on cell survival after 150 kVp X‐rays.^25^ As for the DU145 cell line, the (*a*+*c*) value and the relationship between dose and survival after acute irradiation with 150 kVp X‐rays were obtained in our previous study.^34^ Using these and following steps (i) and (ii), we determined the parameter set [*α*_0_, *β*_0_, (*a*+*c*)] for the DU145 cells. After determining the model parameters, we determined the *h* value using the MCMC method and the experimental survival‐MN relationship,^41^, ^42^, ^45^, ^46^, ^47^, ^48^, ^49^, ^50^, ^51^, ^52^ as the step (iv).

The MCMC simulation enables us to study probability distributions of model parameters, as described previously.^43^ As the first step, the initial set of the model parameters θ ^(0)^ was given before the simulation. We assumed the uniform distributions as the prior distribution for *α*_0_ and *β*_0_, and when performing the MCMC simulation, the candidate parameter set θ ^candidate^ was generated based on the uniform (prior) distributions. Using the experimental survival data *d*, the set of model parameters θ[*α*_0_, *β*_0_, (*a*+*c*)] was sampled using the likelihood *P*(*d_i_* |*θ*) and transition probability *α*_P_ as follows:

(7) $$\begin{array}{cc} P(d|\theta ) & =\prod_{i=1}^{N}\left[P\left(d_{i}|\theta \right)\right]\\ & =\prod_{i=1}^{N}\left\{\frac{1}{\sqrt{2\pi \sigma }}\;\exp\left[-\frac{{\left(-\ln S_{\exp i}+\ln S_{\mathrm{cal}i}\right)}^{2}}{2\sigma^{2}}\right]\right\} \end{array}$$

(8) $$\alpha_{P}=\frac{P\left(\theta^{\mathrm{candidate}}|\;d\right)}{P\left(\theta^{\left(t\right)}|d\right)},$$

where *d_i_* (*i* = 1 *N*) is the measured survival data set, that is, [*d_i_ *= (*D_i_*, −ln *S*_exp_ *_i_*)], *S*_exp_ is the measured surviving fraction, and *S*_cal_ is the surviving fraction estimated by the model. *P*(*θ*|*d*) and *P*(*θ* ^candidate^|*d*) are the posterior likelihood for the candidate (*t*+1)th and previous (*t*)th conditions, respectively. In this study, we set the numbers of burn‐in and sampling of the parameter sets were 10^3^ and 10^4^, respectively. After the MCMC simulation, we obtained the model parameter sets used for predicting cell survival and MN formation in this study. Note that the advantage of the MCMC simulation is that it calculates uncertainties using the sampled parameter sets. The validities of the model parameters for the HSG and SCC cells were evaluated by comparing the predictions to the measured LET dependence of RBE_SF_ for ^12^C beams.^6^, ^41^, ^42^, ^53^, ^54^, ^55^ In the same manner, those for the DU145 and WI‐38 cells were done in comparison with the dose–response curve of surviving fractions.^25^, ^34^

#### 3.1.2 Prediction of LET dependence of MN frequency

Using the determined model parameters, we predicted the dose–response curve and the LET dependencies of MN formation for the HSG and SCC cells. When predicting the dose and LET dependencies, in the same manner as the procedure for determining the model parameters, the y* values for any kinds of ion beams (i.e., C, Ne, Si, Ar, and Fe) were obtained using [t‐sed] tally of PHITS. First, the dose–response curves of the MN frequency for the HSG cells after irradiation with 290 MeV/n carbon beams (LET = 13, 100 keV/µm) were estimated using the model parameters and Equation (3), and were compared to the experimental data.^42^ Second, we simulated the mono‐energy ion beams of carbon, neon, silicon, argon, and iron, and predicted the LET dependencies of the RBE_MN_ for the HSG and SCC cells using Equation (6). The predicted RBE_MN_ values were compared to the experimental RBE_MN_ for various types of heavy ion beams such as C, Ne, Si, Ar, and Fe.^8^, ^53^, ^55^ In the comparison, the uncertainties of RBE_MN_ were also calculated using the model parameter sets sampled by the MCMC simulation.

#### 3.1.3 Prediction of dose‐rate dependence of MN frequency

In addition to the LET dependence described above, we also calculated the dose‐rate dependence of the MN formation frequency. In this calculation, using the model parameters of the WI‐38 and DU145 cell lines, we predicted the relationship between dose rate and the relative MN frequency. Note that the relative MN frequencies were normalized using those at high dose rate (i.e., at 60 Gy/h). The details of our in vitro experiments and protocols are described in the next subsection “3.1.4. Cell culture and MN formation assay” below. Because our in vitro experiments were performed at a constant dose of 4 Gy, so the total absorbed dose in this prediction was also set to be 4 Gy. As with the LET dependence of RBE_MN_, the uncertainty was calculated using the uncertainties of the model parameters.

#### 3.1.4 Cell culture and MN formation assay

We used WI‐38 (IFO50075, Japanese Collection of Research Bioresources Cell Bank, Osaka, Japan) and DU145 (RCB2143, RIKEN BioResearch, Tsukuba, Japan) cell lines. Both the WI‐38 and the DU145 cell lines were grown in RPMI‐1640 with L‐glutamine (Thermo Fisher Scientific Inc.) supplemented with 10% fetal bovine serum (FBS) (Japan Bio Serum, Fukuyama, Japan) and 1% penicillin‐streptomycin (Life Technologies, CA, USA). Both cell lines were maintained at 37°C in a humidified atmosphere of 5% CO_2_ in air.

The cells were exposed to 150 kVp X‐rays (MBR‐1520R‐3; Hitachi Medical Co., Ltd., Tokyo, Japan) at room temperature. For the continuous (acute) irradiation, the maximum dose rate used in this study was 1.0 Gy/min (equivalent to 60 Gy/h). In addition to the acute irradiation at 1.0 Gy/min, we also performed the fractionated irradiation comprised of 0.5 Gy/fraction (Fr) (with 30 s (0.5 min) dose‐delivery time at 1 Gy/min) at 30 s (0.5 min) intervals, 0.1 Gy/Fr (with 6 s dose‐delivery time) at 54 s (0.9 min) intervals, and 0.05 Gy/Fr (with 3 s dose‐delivery time) at 57 s (0.95 min) intervals. Based on the irradiation regimens, we made the average dose rates of 0.5, 0.1, and 0.05 Gy/min, respectively. The total dose was a constant 4 Gy for both cell lines.

The WI‐38 and DU145 cells were seeded in the 4‐well slide chambers (WATSON CO., Ltd., Tokyo, Japan). After 48 h, the seeded cells were irradiated using the X‐ray irradiator following the above irradiation protocols. After 36 h for WI‐38 and 48 h for DU145^56,57^ the cells were fixed in 70% ethanol for 10 min at room temperature and then washed three times with Hanks’ balanced salts solution (FUJIFILM Wako Chemicals, Osaka, Japan). The slides were then stained with 1.0 µg/mL DAPI staining solution (BIO‐RAD, Hercules, CA, USA). The formed MN was scored using confocal laser microscopy (LSM710‐ZEN 2008, Carl Zeiss Micro Imaging Co., Ltd., Jena, Germany). At least 200 cells were counted per culture, and the MN frequency was calculated as the number of MN‐bearing cells/total number of cells. The experiments were repeated at least three times.

### 3.2 Estimates of the surviving fraction from MN data

In the verification of the developed model, we estimated the MN frequency and the RBE_MN_ using the model parameter sets determined by fitting the developed model to cell survival data. Ideally, the RBE_SF_ should also be estimated from the model parameters determined by fitting the model to the dose–response curve concerning the MN frequency. Thus, in addition to the above model validation, we also tried to predict the vice versa approach described above.

Focusing on the HSG cell line because of the extensive data, we determined the model parameters (i.e., *α*_m0_ and *β*_m0_) by fitting the model [Equation (3)] to the experimental dose–response curve on MN formation data.^42^ In this fitting approach, we made the following steps: (i) the lineal energy distributions and *y* ^*^ values for photons and ^12^C ions were calculated using the [t‐sed] tally in PHITS; (ii) the parameter sets [*α*_m0_, *β*_m0_, (*a*+*c*)] were determined by fitting Equation (3) to the experimental MN data^42^ after photon irradiation using MCMC simulation; (iii) the *r*_d_ value was determined by repeatedly comparing the estimated MN with the experimental data for carbon ions.^42^

After determining the model parameters [*α*_m0_, *β*_m0_, (*a*+*c*)] of HSG cells, we estimated the RBE_SF_ based on Equations (4) and (5). It is important to note that the model parameters (*α*_0_ and *β*_0_) were derived from *α*_m0_ and *β*_m0_ using the *h* value determined under the assumption that the *h* value is independent of cell‐line type. Finally, to attempt to validate the *vice‐versa* approach, we estimated the RBE_SF_ values as a function of LET and compared them to the experimental values reported in the literature.^6^, ^42^

### 3.3 Statistics

To determine the significance of induction of the MN frequency depending on the difference in dose rates, the Tukey–Kramer post hoc test after a one‐way analysis of variance was conducted. Statistical analyses were performed using Microsoft Excel 2010 (Microsoft Corporation). In the same manner as the previous experimental report,^25^ *p*‐values below 5% were considered, as significant differences. To evaluate the fit quality of the IMK model developed in this study, we calculated the coefficient of determination *R* ^2^.

## 4 RESULTS

### 4.1 Surviving fraction and the determined model parameters

**FIGURE 2:**
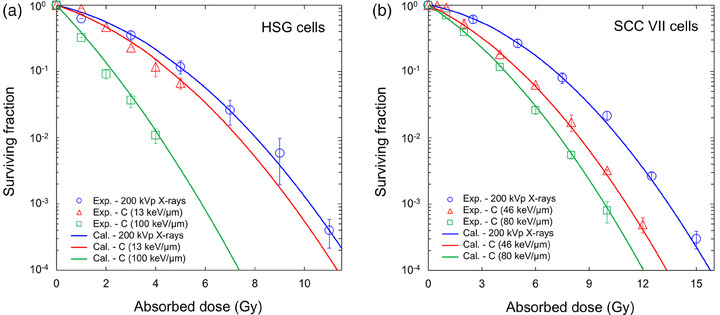
Dose–response curve of cell survival for determining the model parameters. (a) is for the HSG cell line, and (b) is for the SCC VII cell line. The surviving fractions after acute irradiation with 200 kVp X‐rays or the 290 MeV/n carbon beams (LET = 13–100 keV/µm) were estimated using the model parameters in Table 1 and Equation (1), and were compared to the experimental data.^41^, ^42^ HSG, human salivary gland; LET, linear energy transfer.

Figure 2 shows the dose–response curve of cell survival in (a) the HSG and (b) the SCC VII cells. From the fitting, we obtained the parameters for predicting the surviving fraction and the MN frequency. As shown in Figure 2, the IMK model could reproduce the dose–response curves of cell survival not only for photon beams (i.e., 200 kVp X‐rays) but also for the ^12^C ions for various LET. The fitting results of the dose–response curve on cell survival for the DU145 and WI‐38 cell lines are depicted in Figure S3. In addition, the obtained model parameters are listed in Table 1. As seen in Table 1, the uncertainties of the model parameters were obtained by the MCMC simulation. In addition, using the R statistical software (version 4.3.2), the heatmap for each cell line was illustrated in Figure S4. The *r*_d_ and *y*_0_ values for the HSG cells were 0.42 µm and 150 keV/µm, respectively, and were the same as those for the SCC cells. It should be noted that the parameters should depend on the cell line type. In addition, we confirmed that the estimated LET dependence of RBE_SF_ (based on Equation 5) for carbon‐ion irradiation agrees with the corresponding experimental values^6^, ^41^, ^53^, ^54^, ^55^ (see Figure S5).

**TABLE 1:** Model parameters for various cell lines in the IMK model.

Model parameter	Cell line type				unit
	HSG	SCC	WI‐38	DU145	
*α*_0_	1.50 × 10^−1^ ± 6.87 × 10^−2^	8.25 × 10^−2^ ± 4.34 × 10^−2^	5.90 × 10^−1^ ± 1.07 × 10^−1^	7.74 × 10^−2^ ± 3.64 × 10^−2^	Gy^−1^
*β*_0_	4.67 × 10^−2^ ± 7.21 × 10^−3^	2.98 × 10^−2^ ± 3.39 × 10^−3^	1.63 × 10^−2^ ± 1.32 × 10^−2^	3.80 × 10^−2^ ± 4.98 × 10^−3^	Gy^−2^
*a*+*c*	2.19 × 10^0^ ± 4.00 × 10^−1^	2.19 × 10^0^ ± 3.92 × 10^−1^	3.71 × 10^−1^ ± 3.84 × 10^−2^	2.10 × 10^0^ ± 9.11 × 10^−1^	h^−1^
*r*_d_	4.20 × 10^−1^	4.20 × 10^−1^	5.00 × 10^−1^	5.00 × 10^−1^	µm
*h*	2.75 × 10^−1 ^± 6.18 × 10^−3^	2.75 × 10^−1 ^± 6.18 × 10^−3^	2.75 × 10^−1 ^± 6.18 × 10^−3^	2.75 × 10^−1 ^± 6.18 × 10^−3^	–

**FIGURE 3:**
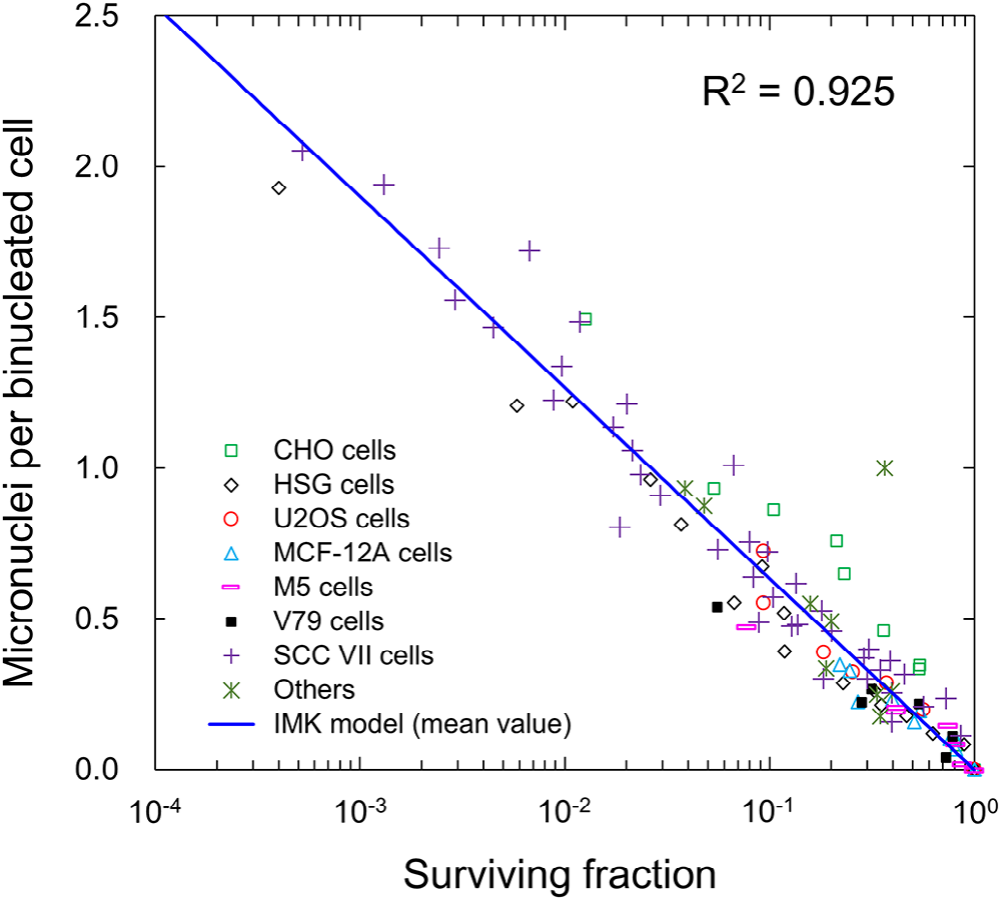
Relation between the surviving fraction and MN per binucleated cell. From the fitting of the model to the experimental relationship,^41^, ^42^, ^45^, ^46^, ^47^, ^48^, ^49^, ^50^, ^51^, ^52^ we calculated the *h* value to be 2.75 × 10^−1 ^± 6.18 × 10^−3^ listed in Table 1. Note that the *h* value for each cell line is summarized in Table 2. MN, micronuclei.

The parameter of *h* was also determined by fitting Equation (3) to the experimental relationship between the surviving fraction and the MN per binucleated cell.^42^, ^43^, ^44^, ^45^, ^46^, ^47^, ^48^, ^49^, ^50^, ^51^, ^52^ Figure 3 depicts the relationship between the log of the surviving fraction and MN frequency per binucleated cell, in which the solid line and the various types of symbols represent the prediction by the IMK model and the experimental data,^42^, ^43^, ^44^, ^45^, ^46^, ^47^, ^48^, ^49^, ^50^, ^51^, ^52^ respectively. Judging from the good agreement using the *R* ^2^ value, the comparison results show that the number of LLs per nucleus <*w*> is intrinsically related to the MN frequency, and it seems to be independent of cell line type. However, it is natural to assume the *h* value should be a cell‐line‐dependent parameter. Assuming that the *h* value depends on the type of cell line, we also obtained the value (mean ± sd) for each cell line, including the CHO, the HSG, U2OS, MCF‐12A, M5, V79, and SCC (VII) cell lines, as summarized in Table 2. The mean values were found to range from 1.96 × 10^−1^ to 3.65 × 10^−1^. The comparison of the distribution of the *h* values is depicted in Figure S6. This means that the *h* value can be dependent on cell‐line type. Meanwhile, considering the uncertainties and *R* ^2^ value in Figure 3, we used the mean value independent of cell line type for predicting the MN frequency in this study.

**TABLE 2:** Model parameter *h* for predicting MN frequency for each cell line.

Cell line type	Radiation type	*h* value		
CHO cells	250 kVp X‐rays, ^125^I	3.65 × 10^−1^	±	3.17 × 10^−2^
HSG cells	200 kVp X‐rays, 290 MeV/u ^12^C ions	2.45 × 10^−1^	±	8.68 × 10^−3^
U2OS cells	^137^Cs γ‐rays	2.61 × 10^−1^	±	3.87 × 10^−2^
MCF‐12A cells	10 kV, 25 kV, 200 kV X‐rays	2.29 × 10^−1^	±	3.72 × 10^−2^
M5 cells	^60^Co γ‐rays, ^6^Li ions	1.96 × 10^−1^	±	4.94 × 10^−2^
V79 cells	^60^Co γ‐rays, ^6^Li ions	1.97 × 10^−1^	±	5.70 × 10^−2^
SCC VII cells	10 MV X‐rays, boron neutron capture therapy	2.81 × 10^−1^	±	7.47 × 10^−3^

Abbreviation: MN, micronuclei.

### 4.2 Dose and LET dependence of MN formation

**FIGURE 4:**
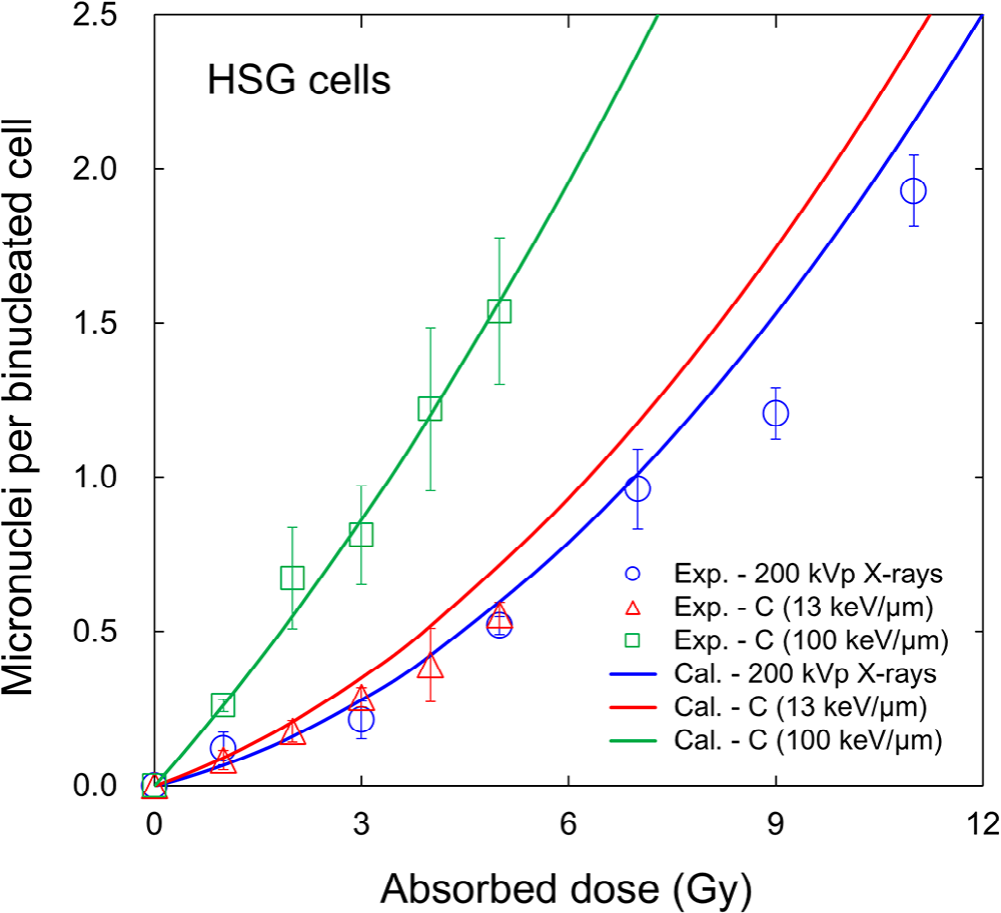
Dose dependence on the MN formation of HSG cells estimated from cell survival data. Using the model parameters listed in Table 1 and Equation (3), we estimated the MN formation in the HSG cell line for X‐rays and 290 MeV/n carbon beams (LET = 13, 100 keV/µm). The model predictions were compared to the corresponding experimental data.^42^ The *R* ^2^ value was 0.974, indicating good agreement between the model prediction and the experimental data. HSG, human salivary gland; MN, micronuclei.

Using the model parameters listed in Table 1, the dose–response curve on the MN formation for the HSG cell line was predicted by the present IMK model. Figure 4 compares the model predictions to the corresponding measured data^42^ of the HSG cells after irradiation with 6‐cm SOBP 290 MeV/n carbon beams (LET = 13, 100 keV/µm). Note that the *y* ^*^ values used for predicting the MN frequency are the same as those used when calculating the surviving fraction shown in Figure 2a. As shown in Figure 4, we observed good agreement between the experimental values and the model predictions using the cell‐line independent *h* value. From the comparison, the dose–response curve on the MN formation follows the LQ relation expressed by Equation (3) of the present IMK model.

**FIGURE 5:**
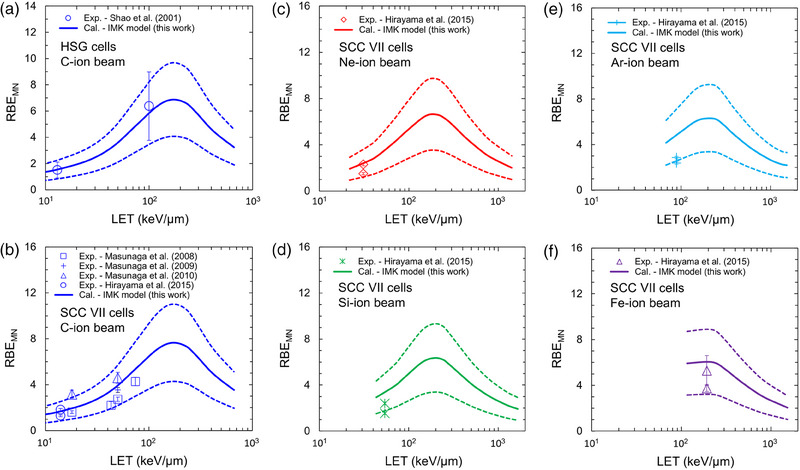
LET dependence of RBE_MN_ for the HSG and the SCC cell lines estimated from cell survival data. (a) and (b) are for the HSG and SCC VII cells irradiated with C ions, respectively, (c) is for the SCC VII cells irradiated with Ne ions, (d) is for Si ions, (e) is for Ar ions, and (f) is for Fe ions. The solid and dotted lines represent the mean value and its uncertainty (i.e., 68% credible interval (CI)), respectively. The model predictions were calculated using the model parameters in Table 1 and Equation (6). Meanwhile, the symbols are the experimental data reported in the literature.^8^, ^42^, ^53^, ^54^, ^55^ Note that when calculating the RBE_MN_, we simulated the mono‐energetic ion beams of carbon, neon, silicon, argon, and iron in the PHITS simulation. HSG, human salivary gland; RBE, relative biological effectiveness; SCC, squamous cell carcinoma.

In addition to the dose–response curve on the MN formation (Figure 4), we also compared the model predictions to the experimental RBE_MN_ for various types of heavy ions. (i.e., C, Ne, Si, Ar, and Fe).^8^, ^42^, ^53^, ^54^, ^55^ Figure 5 shows the comparison results between the IMK model prediction and the experimental LET‐dependencies of RBE_MN_, where (a) and (b) are for the HSG and SCC VII cells irradiated with C ions, respectively, (c) is for the SCC VII cells irradiated with Ne ions, (d) for Si ions, (e) for Ar ions, and (f) for Fe ions. In Figure 5, the solid and dotted lines represent the mean value and its uncertainty (i.e., 68% credible interval (CI)), respectively. The experimental radiation qualities are made by changing the depth or radiation energy, so it is difficult to completely reproduce the irradiation geometry in the PHITS simulation. Therefore, by simulating the mono‐energetic heavy‐ion beams, we calculated the LET dependence of RBE_MN_ in this study. From Figures 4 and 5, considering the uncertainties of model prediction, it is shown that the present IMK model is useful to predict the LET dependence of not only RBE_SF_ but also RBE_MN_. Meanwhile, the amount of available experimental data is limited, as shown in Figure 5. Although the cumulation of the experimental RBE_MN_ for high‐LET irradiation is required, the RBE_MN_ values in the LET range of < a few tens of keV/µm, which is clinically used, were fairly reproduced by the present IMK model (see Figure 5b).

### 4.3 Dose‐rate dependence of the MN formation

The developed model was verified compared to the dose and LET dependence of the MN frequency. To further verify the model, focusing on the different rates of SLD repair of normal and cancer cell lines, WI‐38 and DU145, we next measured the dose‐rate dependence of the MN formation and compared the model with the experimental data.

**FIGURE 6:**
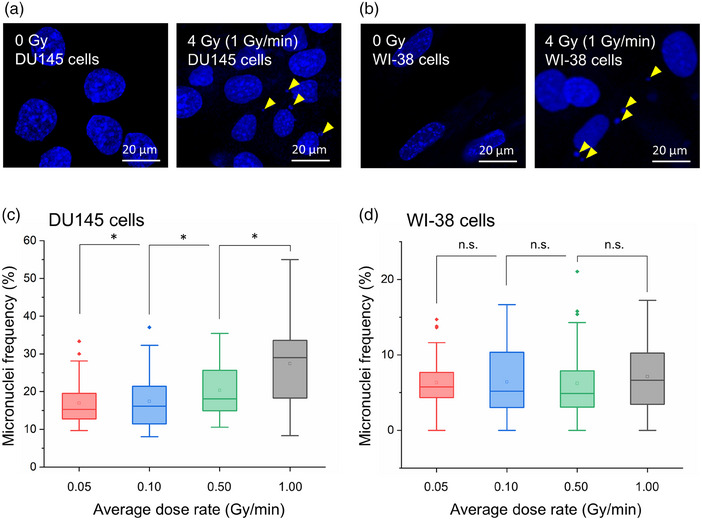
Microscopic images of the MN frequency and measured dose‐rate dependence. (a) is the DU145 and (b) is the WI‐38 cells, in which both cell lines were irradiated with a total dose of 4 Gy. The MN frequency in the DU145 cell line decreases with decreasing dose rate. Meanwhile, there seems to be less dose‐rate dependence in the WI‐38 cells. The symbols * and n.s. represent significant differences compared to 1 Gy/min and no significance, respectively. MN, micronuclei.

**FIGURE 7:**
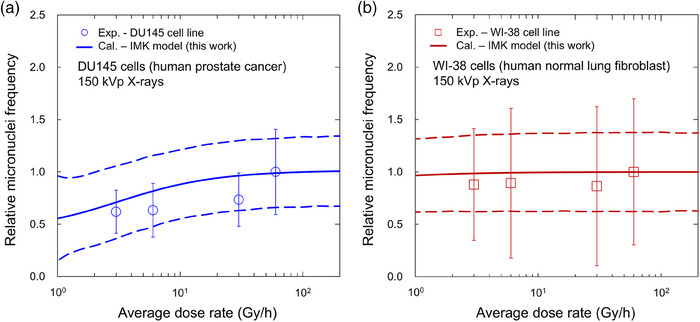
Prediction of the dose‐rate dependence of the MN frequency estimated from cell survival data. (a) is the DU145, and (b) is the WI‐38 cells. In the same manner as Figure 6, both cell lines were irradiated with a total dose of 4 Gy. The solid and dotted lines represent the mean value and the 95% CI, respectively. Similar to the experimental results, the model prediction exhibits a decrease in the DU145 cells and no dose‐rate dependence in the WI‐38 cells. The *R* ^2^ value was 0.707, showing a good agreement between the model prediction and the experimental data. MN, micronuclei.

Figure 6 shows the microscopic images of the MN frequency in (a) the DU145 and (b) the WI‐38 cells, in which both cell lines were irradiated with 4 Gy at various dose rates. The fraction of the MN frequency is depicted in Figure 6c,d. Although the fraction of the MN frequency in the DU145 cell line significantly decreases with decreasing dose rate, there is no significant dose‐rate dependence in the WI‐38 cells. Not that the * and n.s. represent significant differences compared to 1 Gy/min and the no significance, respectively. This is because the SLD repairs of both cell lines are different from each other (i.e., 2.10 × 10^0^ ± 9.11 × 10^−1^ for the DU145 cell line and 3.71 × 10^−1^ ± 3.84 × 10^−2^ for the WI‐38 cell line). Using these model parameters, including the SLD repair rate (*a*+*c*), we predicted the MN frequency relative to the high dose rate (i.e., 1 Gy/min), as shown in Figure 7. In the same manner as Figure 5, the solid and dotted lines represent the mean value and its 95% CI, respectively. The model prediction also exhibits a decrease in the DU145 cells and no dose‐rate dependence in the WI‐38 cells. From the comparison in Figure 7, we verified that the model enables the prediction for dose, LET, and dose‐rate dependencies of the MN frequency, suggesting that the MN formation is intrinsically related to LL formation and cell survival.

### 4.4 Prediction of RBE_SF_ from MN frequency data

In addition to the RBE_MN_ prediction (Figure 5), it is ideal that the RBE_SF_ can also be predicted by the model parameters determined from the dose–response curve of MN frequency. Considering this, to further verify the model performance, we also applied the developed model (i.e., Equation (3)) to the relationship between dose and the MN frequency, and tried to estimate the LET dependence of RBE_SF_. Note that the model parameters were determined by the MCMC simulation in the same manner as the determination methods for the model parameters listed in Table 1.

**FIGURE 8:**
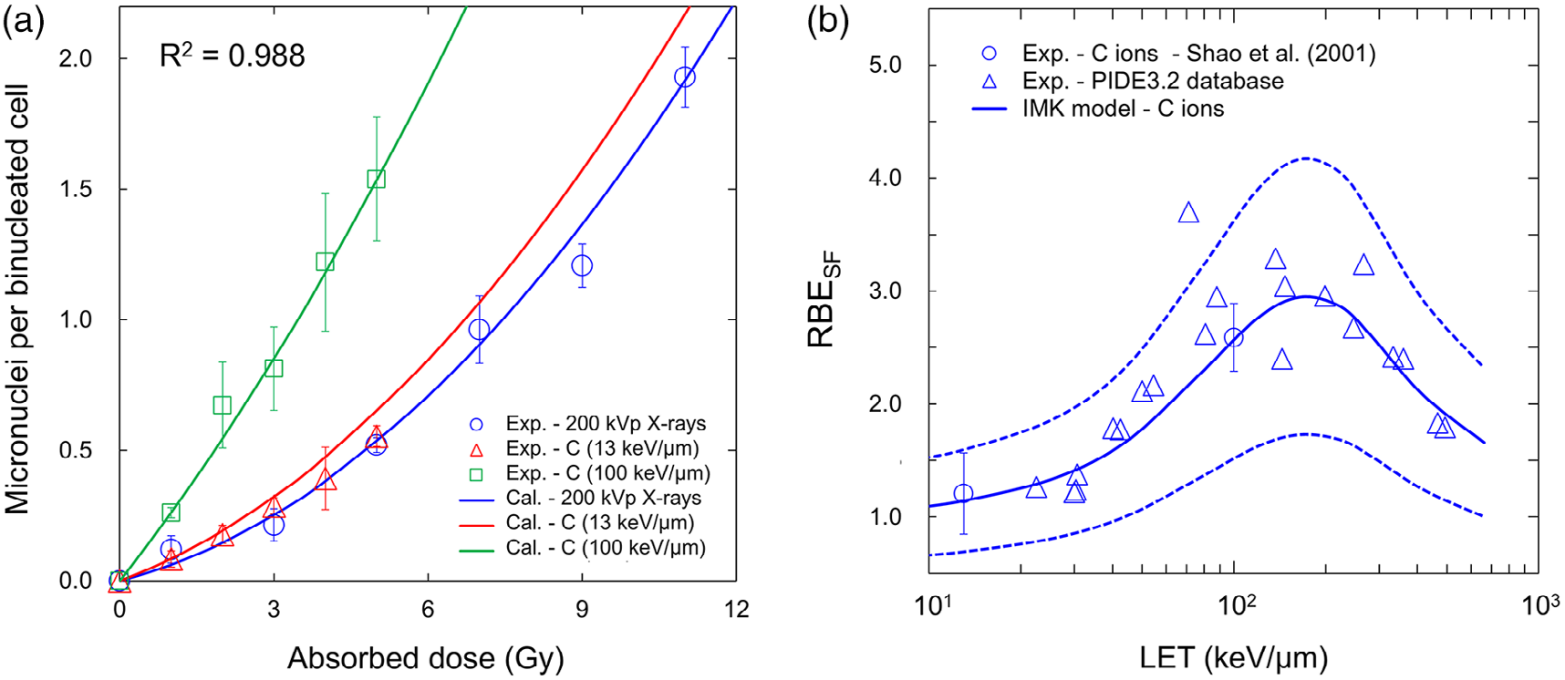
Model predictions of RBE_SF_ from the fitting to the MN frequency data. (a) is the dose–response curve on MN frequency and (b) is the LET dependence of RBE for survival. The model parameters used for this prediction, that is, *θ*_MN_(*α*_m0_, *β*_m0_, *a*+*c*, *r*_d_) = (3.75 × 10^−2^ ± 3.28 × 10^−2^ [Gy^−1^], 1.13 × 10^−2^ ± 3.48 × 10^−3^ [Gy^−2^], 2.19 × 10^0^ ± 3.91 × 10^−1^ [h^−1^], 0.40 [µm]), were different from those listed in Table 1. The model predictions (solid line) were compared to the corresponding experimental data (symbols). Note that the dotted lines represent the 68% CI calculated using the parameters’ uncertainties.

Figure 8a shows the fitting results of the IMK model to the experimental MN data.^42^ The set of the model parameters was found as follows: *θ*_MN_(*α*_m0_, *β*_m0_, *a*+*c*, *r*_d_) = (3.75 × 10^−2^ ± 3.28 × 10^−2^ [Gy^−1^], 1.13 × 10^−2^ ± 3.48 × 10^−3^ [Gy^−2^], 2.19 × 10^0^ ± 3.91 × 10^−1^ [h^−1^], 0.40 [µm]). Because Figure 8a represents the fitting results, the prediction curves show better agreement compared to those in Figure 4. Using the model parameters *θ*_MN_, the LET dependence of RBE_SF_ was predicted. As shown in Figure 8b, the present IMK model agrees well with the corresponding measured values.^6^, ^42^ Particularly, most of the measured data were found to be within the bounds of prediction uncertainties. It should be noted that the dotted lines represent the 68% CI calculated using the uncertainties of the model parameters *θ*_MN_. Figure S7 compares the predicted dose response curve on cell survival with the experimental values, which also shows good agreement. Therefore, it was proven that the developed model is effective even for predicting RBE values for cell survival from the MN‐frequency data.

## 5 DISCUSSIONS

By introducing the new parameter *h* in the IMK model, we extended the IMK model^20^, ^21^ to predict the MN formation frequency (see section: “*Overview of the IMK model*”). In this study, assuming that the *h* value is independent of cell line type (see Figure 3), we deduced the *h* parameter from the experimental relation between cell survival and the MN per binucleated cell.^41^, ^42^, ^45^, ^46^, ^47^, ^48^, ^49^, ^50^, ^51^, ^52^ Meanwhile, the formation of the MN can be intrinsically related to the unrepaired (mis‐repaired) DSBs.^9^ There are two major pathways of DSB repair, the NHEJ and the homologous recombination (HR).^58^ The repair process in the model is interpreted as NHEJ,^59^ which is the faster repair compared to the HR.^60^ The contents of the MN are often fragmented and ligated by NHEJ.^61^ However, the ability of the NHEJ depends on the cell cycle^62^ and is eventually dependent on the cell type. Considering these factors, it is natural to assume that the *h* parameter should depend on the cell‐line type (as evaluated in Figure S6 in the supplementary material) as well as NHEJ‐ or HR‐deficient cells. However, to ascertain whether or not the *h* value should be cell‐line dependent, it is essential that future studies conduct further evaluation through the accumulation of experimental data.

When evaluating the radiation‐induced MN formation using the IMK model (see Figures 4, 5, and 7), we used the different types of MN frequency data (i.e., MN per binucleated cell and MN fraction in cell population). As for the issue of experimental techniques, the MN frequency can vary depending on the assay type, meaning that the sensitivity of the *h* largely depends on the experimental protocol. Considering this, when predicting the MN frequency as an absolute value, it is necessary to unify the experimental protocol and to re‐determine the *h* parameter. In other words, we do not recommend using the *h* value reported in this paper as is. To evaluate the influence of MN assay protocols on determining the *h* value, it is needed to accumulate the MN data using the same cell line. Meanwhile, as concluded in this study, radiation‐induced MN is intrinsically related to the LL formation and cell killing after radiation exposure (see Figure 2). Therefore, we believed that the relative value of MN frequency (i.e., RBE_MN_) can be predicted without using the *h* parameter. Regarding this, its estimation can be useful for radiosensitivity analysis in the fields of radiation protection and radiation therapy. Particularly, targeted alpha therapy (TAT)^63^, ^64^ has been followed with increasing interest in radiation therapy; however, it is expected to be difficult to measure the clonogenicity because several biological factors (e.g., cell cycle) can be important during the protracted irradiation.^21^ In some situations, the protracted exposure at a low dose rate potentially induces inverse dose protraction effects.^65^, ^66^ Compared to the conventional colony assay,^3^ the MN assay is useful for evaluating radiosensitivity in less time. As shown in Figure 8b, the RBE_SF_ values were also found to be calculated from the model prediction using the MN frequency data. Considering that its ability to predict cell survival from MN frequency and vice versa, we can conclude that the MN formation assay can be an alternative method to determine the RBE value for cell killing in radiation protection and radiation therapy.

Because of its rapidness, simplicity, and potential for automation, the measurement of the MN micronucleated cells is believed to be a sensitive biological indicator of clastogenic effects (chromosome aberrations).^10^, ^67^ In accordance with this, the present theoretical study clearly demonstrates that there is an intrinsic link between MN formation and cell death (see Figures 4, 5, and 7). We interpreted that this is because cells with MN containing chromosome fragments are expected to be unstable, leading to cell death.^68^ In other words, the MN formation assay is useful as biodosimetry to assess genotoxicity.^12^ One report concluded that apoptosis might contribute to the elimination of micronucleated cells,^69^ which agrees well with the scenario (assumption) made in the present IMK model (see section: “*Overview of the IMK model*”). Meanwhile, it is reported that some of the micronucleated cells may survive and pose a carcinogenic risk.^70^ In fact, it has been reported that there is a good relationship between the log of surviving fractions (equivalent to LLs) and mutation frequency.^71^ Considering these facts, the present model has the possibility to extend to the prediction of chromosome aberrations and carcinogenesis in the future. However, in the current state, the available experimental data for developing the prediction model are insufficient and limited. In particular, the dependence of the cell type and dose rate on the *h* parameter cannot be discussed in any more detail than in this paper at this stage due to the limited data available. Further accumulation of in vitro and in vivo data for making scenarios in prediction models is essential in the future.

## 6 CONCLUSIONS

This work presents a biophysical model for estimating micronuclei (MN) frequency by extending the IMK model. The developed model could successfully reproduce the experimental dose, LET, and dose‐rate dependencies of the MN formation and its relative biological effectiveness (RBE_MN_), indicating that radiation‐induced MN is intrinsically related to the lethal lesion formation and cell killing after radiation exposure. Through the present model analysis, we confirmed that the RBE for cell survival and MN frequency are equivalent under the same irradiation conditions. Based on these results, this modeling study suggests that the measurement of MN frequency is useful in both radiation therapy and protection when quantitatively evaluating radiosensitivity at early stages after exposure. In addition, the IMK model includes various radiosensitive parameters to reproduce the cellular responses to date, suggesting the possibility of predicting the MN frequency for various irradiation situations. However, the available experimental data are still limited. Therefore, further accumulation of experimental MN data and further model verification (including development) are necessary in the future.

## CONFLICT OF INTEREST STATEMENT

The authors declare that they have no conflict of interest.

## Supporting information



Supporting Information
