# Supplementary material for: Equivalent relative biological effectiveness for cell survival and micronuclei formation: insights from a biophysical approach
Source: Med Phys. 2025 Sep 30;52(10):e70040. doi: 10.1002/mp.70040 (PMC12483971; doi:10.1002/mp.70040)
Supplement: Supplementary file 1 — Supporting Information [file MP-52-0-s001.pdf]

Supplementary Material: "*Equivalent Relative Biological Effectiveness for Cell Survival and Micronuclei Formation: Insights from A Biophysical Approach*"

In this study, we developed a biophysical model, the integrated microdosimetric-kinetic (IMK) model[1,2], to predict the frequency of micronuclei (MN) formation and its relative biological effectiveness (RBE). This supplementary file includes six figures: Figure S1: Ridge filter considered in the PHITS code; Figure S2: Dose distribution and LET for 6-cm SOBP carbon ions; Figure S3: Cell survival curve in DU145 and WI-38 cells; Figure S4. Heatmap of the parameters in the IMK model; Figure S5: LET dependence of RBE for cell survival after irradiation with C-ion beams; Figure S6: Comparison of *h* value distribution of each cell line; and Figure S7: Cell survival curve of HSG cells estimated from the MN data.

I. *PHITS simulation for calculating the lineal-energy distribution*

To obtain the model parameters (Table 1 in the main manuscript), we fitted the developed IMK model to the experimental cell survival curve[3,4] irradiated with 6-cm spread-out Bragg peak (SOBP) carbon ions. In this fitting procedure, we demonstrated the experimental irradiation geometry of the carbon-ion beams and calculated the lineal-energy distributions for the SOBP carbon ions using a Monte Carlo simulation code for radiation transport, named Particle and Heavy Ion Transport code System (PHITS).[5]

To demonstrate the experimental geometry of the carbon-ion beams[3,4], we used the lecture folder in the PHITS package (phits/lecture/therapy/RidgeFilter). Figure S1 shows the shape of the ridge filter, which was designed to reproduce the experimental percentage of depth dose (PDD) reported in the previous report.[3] To calculate the dose distribution, we used the [t-deposit] tally, which allows us to obtain energy deposition in certain regions. In the same manner, we also calculated the dose-averaged Linear Energy Transfer (LET) for the 6-cm SOBP using the [t-LET] tally that provides the information on track length and dose as a function of the LET. Using the obtained results, we determined the depth position of the 6-cm SOBP carbon irradiation to reproduce the experimental dose-averaged LET.[3,4]

Figure S2 compares the PDD simulated by the PHITS code (the black solid line) and the measured values (the black dotted line), where the dose-averaged LET is also depicted using the blue solid line. To calculate the PDD and the LET, the cutoff energy of carbon ions was set to be 1 keV/n. As shown in Fig. S2, the PDD calculated by the PHITS code agrees well with the experimental results.[3,6] From the good agreement, we confirmed that the irradiation geometry considered in the PHITS code enables the demonstration of the experimental irradiation and the evaluation of the microscopic distribution, i.e., the lineal-energy (y) distribution. Note that the y

distributions were calculated using the [t-sed] tally, which can compute the distribution of energy deposited in the domain in the macroscopic Monte Carlo simulation.

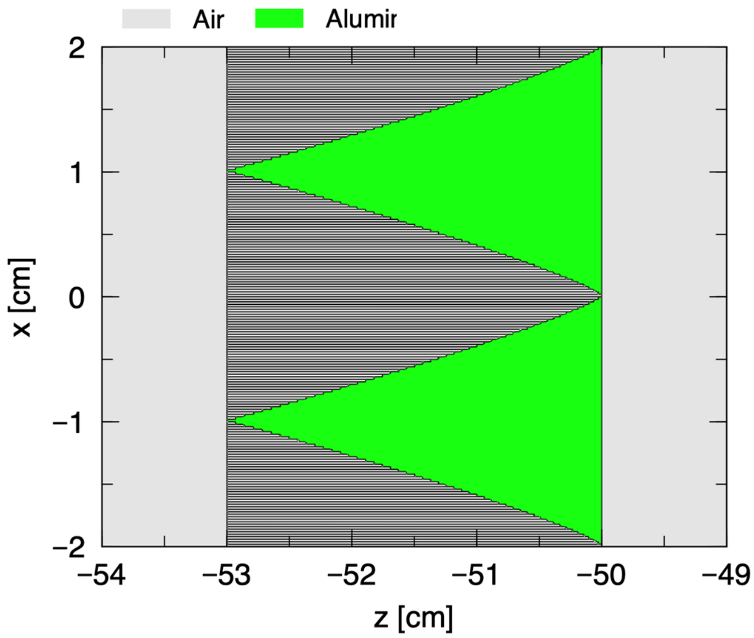

Figure S1. Ridge filter considered in the PHITS code. The shape of the ridge filter, which was designed to reproduce the experimental percentage of depth dose (PDD) reported in the previous report.[3] Note that to demonstrate the experimental geometry of the carbon-ion beams[3,4], we used the lecture folder in the PHITS package (phits/lecture/therapy/RidgeFilter).

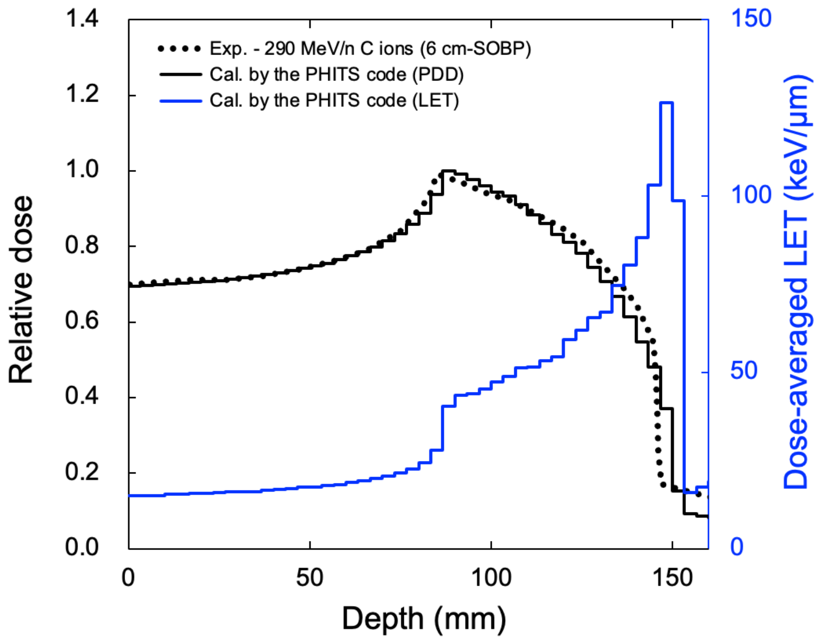

Figure S2. Dose distribution and LET for 6-cm SOBP carbon ions. The black solid line and the dotted line represent the PDD simulated by the PHITS code and the measured values[3,6], respectively. The blue solid line means the dose-averaged LET. The dose and the LET were calculated using the [t-deposit] and [t-LET] tallies.

II. *Estimation of the cell survival and the determined model parameters*

In the main paper, we fitted the developed IMK model to the experimental dose-response curves on cell survival,[3,4,8,9] and obtained the model parameters of HSG, SCC, DU145, and WI-38 cell lines (see Table 1). In the fitting approach, we used the Markov chain Monte Carlo (MCMC) method to obtain the set of the model parameters. In this supplementary material, we show the fitting results of the cell survival curve in the DU145 and the WI-38 cell lines.

Figure S3 depicts the dose-response curve of the surviving fraction after exposure to 150 kVp X-rays, where solid lines and symbols represent the prediction by the IMK model and the experimental data, respectively.[8,9] The dose rate of the 150 kVp X-rays was 1.82 Gy/min. As shown in Fig. S3, we confirmed that the IMK model agrees well with the experimental data. The model parameters of the DU145 and the WI-38 cells are summarized in Table 1. The parameter sets of the HSG and the SCC cells are also listed in Table 1 and the predicted survival curves are depicted in Fig. 2 in the main paper. By virtue of the MCMC simulation, we obtained the uncertainties of the model parameters.

Figure S4 shows the heatmap of the frequency of model parameters (α_0, β_0) of four types of cell lines, i.e., (a) HSG, (b) SCC VII, (c) DU145, and (d) WI-38. Using the sets of the model parameters, we calculated the uncertainties of $\mathrm{RBE}_{\mathrm{MN}}$ (shown in Fig. 5 in the main paper) and those of relative MN frequency as a function of dose rate (shown in Fig. 7 in the main paper).

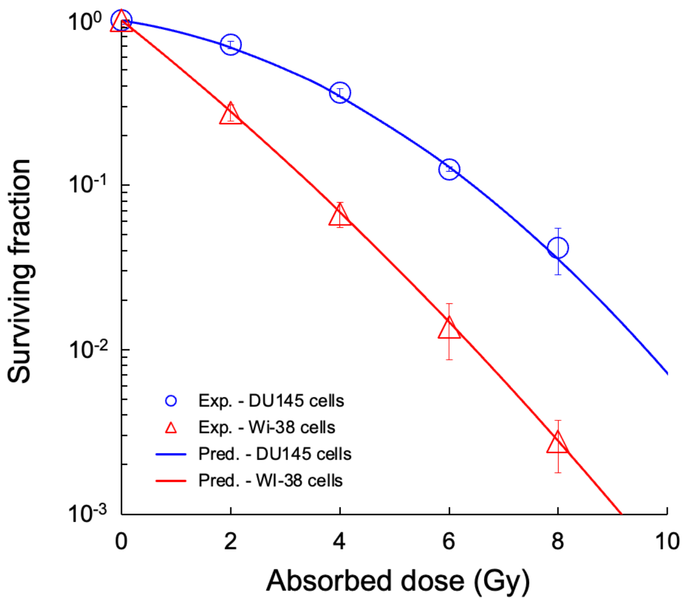

Figure S3. Cell survival curve in DU145 and WI-38 cells. Using the experimental survival data[8,9], we performed the MCMC simulation to obtain the set of the model parameters of the DU145 and WI-38 cell lines. The model parameters are summarized in Table 1 in the main paper.

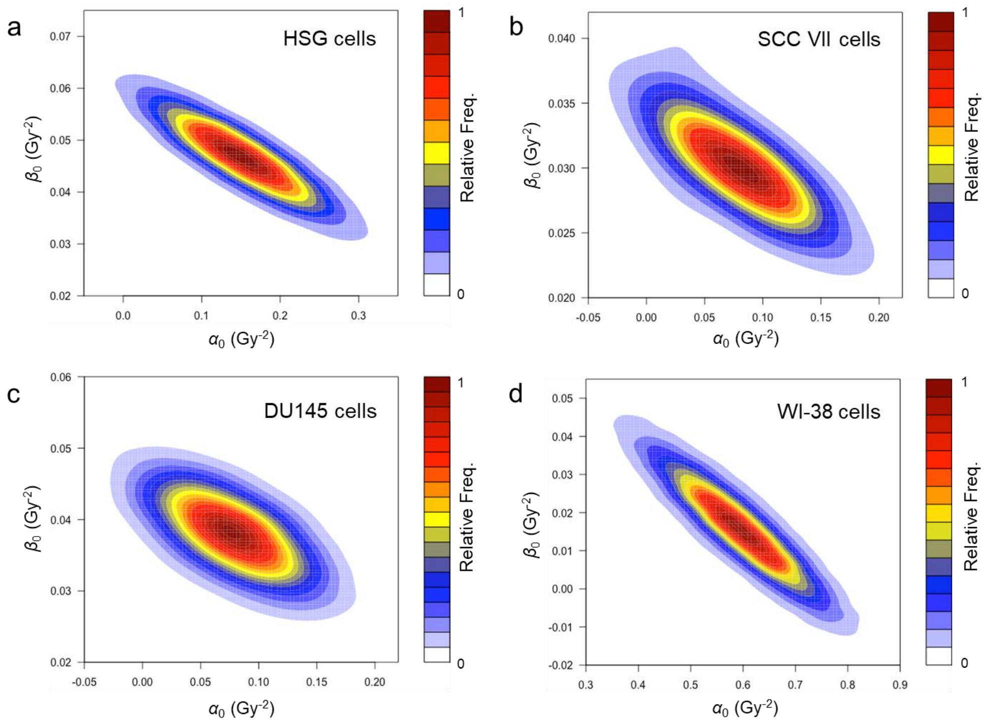

Figure S4. Heatmap of the parameters in the IMK model. (a) is the map for the HSG cells, (b) is for the SCC VII cells, (c) is for the DU145 cells, and (d) is for the WI-38 cells. To depict the map, we performed the MCMC simulation and obtained the sets of the model parameters for each cell line. We made these heatmaps using the MASS (Modern Applied Statistics with S) package (version 7.3-61) for R statistical software (version 4.3.2).

III. *Benchmark tests of the IMK model for surviving fraction*

In the results section in the main paper, we firstly used the developed IMK model to predict the MN frequency, the RBE_{MN}, and relative frequency as a function of dose rate. The validation of the model for predicting cell survival was also shown in Fig. 2 in the main paper. However, the comparisons between the model prediction and the experimental dose-response curve of surviving fraction in Fig. 2 are insufficient. Considering this, we estimated the RBE_{SF} based on Eq. (5) expressed in the main paper, and compared the predicted RBE_{SF} with the corresponding experimental values.[3,10-13]

Figure S5 depicts the LET dependence of RBE for cell survival after irradiation with C-ion beams, where (a) is for the HSG cells and (b) is for the SCC cells. Whilst the symbols represent the experimental data taken from literatures[3,10-13], the solid and dotted lines were the mean prediction values and the 95% credible interval (CI) corresponding to the double standard deviations (2sd), respectively. In addition to Fig. 2, as shown in Fig. S5, the IMK model exhibits the increase of the RBE values with increasing LET and reaches to peak at 150 keV/μm (which means the over-kill effects). The tendency is similar to the experimental data. Taking account of the prediction uncertainties, the IMK model agrees well with the experimental data.

Therefore, we confirmed that the IMK model enables the reproduction of both RBE values for both MN frequency and the surviving fraction.

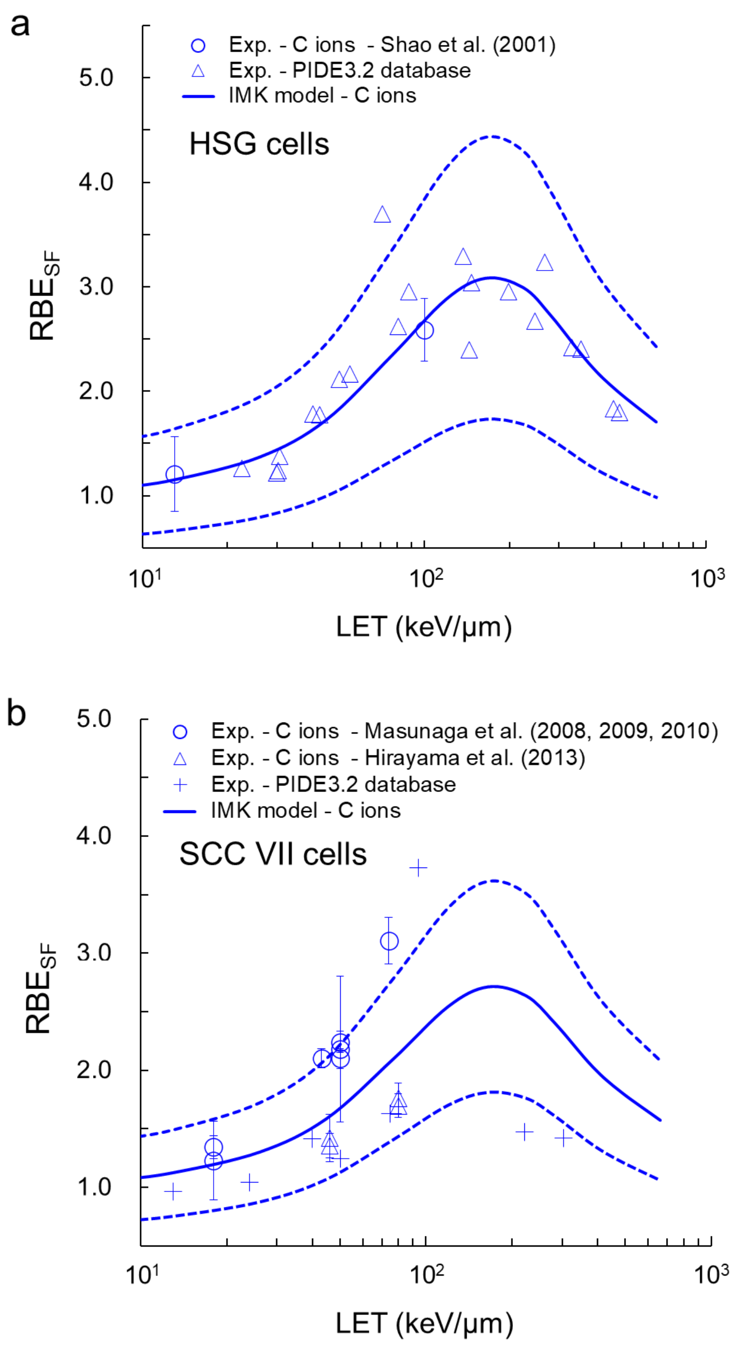

Figure S5. LET dependence of RBE for cell survival after irradiation with C-ion beams. (a) is for the HSG cells and (b) is for the SCC cells. The symbols represent the experimental data taken from the literature.[10-13] Meanwhile, the solid and dotted lines represent the mean prediction values and the 95% CI (corresponding to 2 s.d.), respectively. The prediction curves were estimated using the model parameters determined from the cell survival data. Therefore, we confirmed that the IMK model enables the reproduction of both RBE_{SF} values.

IV. *Evaluation of the h parameter for each cell line*

We assumed that the h value is independent of the cell-line type and estimated the MN frequency in the main paper (see Fig. 3). However, as discussed in the main paper, the formation of the MN can be intrinsically related to the unrepaired DNA double-strand breaks (DSBs).[14] The contents of the MN are often fragmented and ligated by the faster repair pathway of DSBs, i.e., non-homologous end joining (NHEJ).[15] The ability of the NHEJ depends on cell cycle[16] and is eventually dependent on cell type. In other words, it is natural to consider that the h parameter should depend on the cell-line type.

Considering these, we also evaluated the h value for each cell line, including the CHO, HSG, U2OS, MCF-12A, M5, V79, and SCC (VII) cell lines. Table 2 of the main paper lists the mean value and the standard deviations. In this supplementary material, in addition to Table 2, we evaluated the uncertainties of the h values for 7 types of cell lines by using the value obtained from the MCMC simulations. Figure S6 compares the h distributions for various cell line types, which were depicted using the MASS (Modern Applied Statistics with S) package (version 7.3-61) for R statistical software (version 4.3.2). As shown in Fig. S6, the peak values and the ranges seem to be varied among the cell line types. As described in the main paper, from Fig. S6, this means that the h value can be dependent on cell-line type.

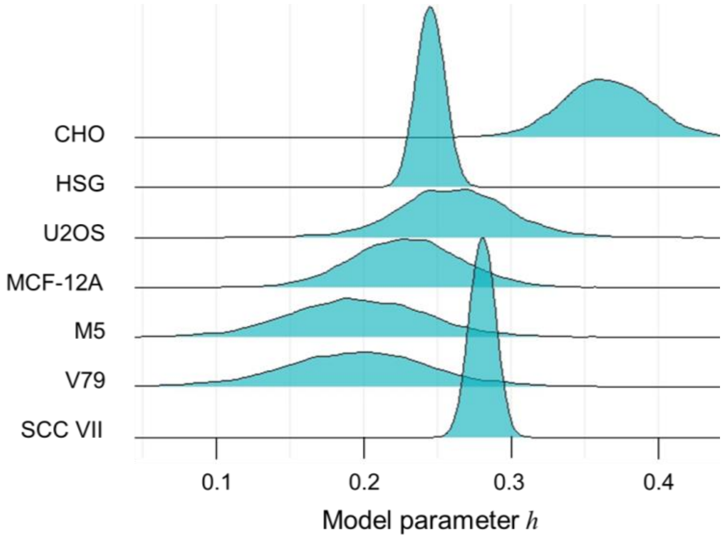

Figure S6. Comparison of *h* value distribution of each cell line. The distribution (histograms) of the h values for 7 types of cell lines were depicted using the results of the MCMC simulations. We compared the distributions using the MASS (Modern Applied Statistics with S) package (version 7.3-61) for R statistical software (version 4.3.2).

Meanwhile, judging from the uncertainties and the R^2 value in Fig. 3, it seems to be also sufficient to use the cell-type independent value. Generally speaking, from the discussion in the main paper, it is natural to consider that the h parameter should depend on the cell-line type;

however, to conclude whether the h value should be cell-line dependent or not, further evaluation through the accumulation of experimental data is essential in future study.

V. *Dose-response curve on cell survival from the fitting to the MN data*

In the main paper, we attempted to estimate the LET dependence of RBE_{MN} using the model parameters obtained by fitting the model to experimental dose-response data on cell survival. As described in the main paper, ideally, the dose-response curve on cell survival should also be estimated from the model parameters determined by fitting the model to the dose-response curve concerning MN frequency. In this supplemental material, we also show the dose-response curves on cell survival estimated by the *vice-versa* approach.

Figure S7 compares the predicted dose-response curve on cell survival (solid line) with the experimental values (symbols). To estimate the surviving fraction, we used the set of model parameters as follows: $\theta_{MN}(\alpha_{m0}, \beta_{m0}, a+c, r_d)$ = ($3.75\times10^{-2} \pm 3.28\times10^{-2}$ [Gy^{-1}], $1.13\times10^{-2} \pm 3.48\times10^{-3}$ [Gy^{-2}], $2.19\times10^{0} \pm 3.91\times10^{-1}$ [h^{-1}], 0.40 [μm]). As shown in Fig. S7, the model prediction agrees well with the corresponding experimental value judged from R^2 = 0.985. In the main paper, Fig. 8b shows the good agreement between the predicted LET dependence of RBE_{SF} and the corresponding experimental value, indicating that the developed model is effective even for predicting cell survival from the MN-frequency data.

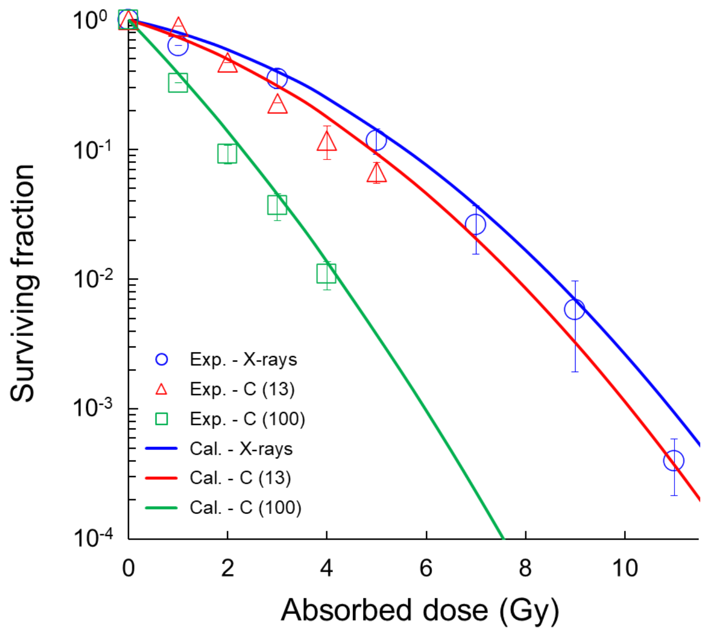

Figure S7. Cell survival curve of HSG cells estimated from the MN data. The set of the model parameters were found as follows: $\theta_{MN}(\alpha_{m0}, \beta_{m0}, a+c, r_d)$ = ($3.75\times10^{-2} \pm 3.28\times10^{-2}$ [Gy^{-1}], $1.13\times10^{-2} \pm 3.48\times10^{-3}$ [Gy^{-2}], $2.19\times10^{0} \pm 3.91\times10^{-1}$ [h^{-1}], 0.40 [μm]). The model predictions were compared to the corresponding experimental data.[4] The R^2 value was 0.985, indicating a good agreement between the model prediction and the experimental data.

References

1. Y. Matsuya, H. Fukunaga, M. Omura, H. Date. A Model for Estimating Dose-Rate Effects on Cell-Killing of Human Melanoma after Boron Neutron Capture Therapy, *Cells* 9(5) (2020) 1117.

2. Y. Matsuya, S.J. McMahon, K. Tsutsumi, K. Sasaki, G. Okuyama, Y. Yoshii, R. Mori, J. Oikawa, K.M. Prise, H. Date. Investigation of dose-rate effects and cell-cycle distribution under protracted exposure to ionizing radiation for various dose-rates, *Sci. Rep*. 8 (2018) 8287.

3. R. Hirayama, A. Uzawa, N. Takase, Y. Matsumoto, M. Noguchi, K. Koda, M. Ozaki, K. Yamashita, H. Li, Y. Kase, N. Matsufuji, S. Koike, S. Masunaga, K. Ando, R. Okayasu, Y. Furusawa, Evaluation of SCCVII tumor cell survival in clamped and non-clamped solid tumors exposed to carbon-ion beams in comparison to X-rays, *Mutat. Res*. 756(1–2) (2013) 146–151.

4. C. Shao, M. Aoki, Y. Furusawa. Medium-mediated Bystander Effects on HSG Cells Co-cultivated with Cells Irradiated by X-rays or a 290 MeV/u Carbon Beam, *J. Radiat. Res*. 42(3) (2001) 305–316.

5. T. Sato, Y. Iwamoto, S. Hashimoto, T. Ogawa, T. Furuta, S. Abe, T. Kai, Y. Matsuya, N. Matsuda, Y. Hirata, T. Sekikawa, L. Yao, P.E. Tsai, H.N. Hunter, H. Iwase, Y. Sakaki, K. Sugihara, N. Shigyo, L. Sihver and K. Niita. *J. Nucl. Sci. Technol.*, 2024, **61**, 127–135.

6. S. Ando. K. Koike. Y.-J. Nojima, C. Chen. S. Ohira. N. Ando T. Kobayashi, W. Ohbuchi, T. Shimizu. K. Kanai. Mouse skin reactions following fractionated irradiation with carbon ions. *Int. J. Radiat. Biol.* 74(1) (1998) 129–138.

7. T. Sato, R. Watanabe, Y. Kase, C. Tsuruoka, M. Suzuki, Y. Furusawa, K. Niita. Analysis of cell-survival fractions for heavy-ion irradiations based on microdosimetric kinetic model implemented in the particle and heavy ion transport code system, *Radiat. Prot. Dosim.* 143 (2011) 491–496.

8. Y. Matsuya, T. Sato, Y. Yachi, H. Date, N. Hamada. The impact of dose rate on responses of human lens epithelial cells to ionizing irradiation, *Sci. Rep.* 14 (2024) 12160.

9. Y. Matsuya, S.J. McMahon, K.T. Butterworth, Y. Yachi, R. Saga, T. Sato, K.M. Prise. Modelling oxygen effects on the in- and out-of-field radiosensitivity of cells exposed to intensity-modulated radiation fields, *Phys. Med. Biol.* 68 (2023) 095008.

10. T. Friedrich, T. Pfuhl, M. Scholz. Update of the particle irradiation data ensemble (PIDE) for cell survival, *J. Radiat. Res*. 62(4) (2021) 645–655.

11. S. Masunaga, K. Ando, A. Uzawa, R. Hirayama, Y. Furusawa, S. Koike, K. Ono. The radiosensitivity of total and quiescent cell populations in solid tumors to 290 MeV/u carbon ion beam irradiation in vivo. *Acta Oncol.*47(6) (2008) 1087–1093.

12. S. Masunaga, R. Hirayama, A. Uzawa, G. Kashino, M. Suzuki, Y. Kinashi, Y. Liu, S. Koike, K. Ando, K. Ono. The effect of post-irradiation tumor oxygenation status on recovery from radiation-induced damage in vivo: with reference to that in quiescent cell populations. *J. Cancer Res. Clin. Oncol*. 135(8) (2009) 1109–1116.

13. S. Masunaga, R. Hirayama, A. Uzawa. et al. Influence of manipulating hypoxia in solid tumors on the radiation dose-rate effect in vivo, with reference to that in the quiescent cell population. *Jpn. J. Radiol.* 28 (2010) 132–142.

14. K. Krupina, A. Goginashvili, D.W. Cleveland. Causes and consequences of micronuclei, *Cur. Opinion Cell Biol.* 70 (2021) 91–99.

15. Y. Oobatake, N. Shimizu. Double-strand breakage in the extrachromosomal double

minutes triggers their aggregation in the nucleus, micronucleation, and morphological transformation. *Genes Chromosomes Cancer* 59(3) (2020) 133–143.

16. Z. Mao, M. Bozzella, A. Seluanov, V. Gorbunova. DNA repair by nonhomologous end joining and homologous recombination during cell cycle in human cells. *Cell cycle* 7(18) (2008) 2902–2906.
